# A Guide for Water Bolus Temperature Selection for Semi-Deep Head and Neck Hyperthermia Treatments Using the HYPERcollar3D Applicator

**DOI:** 10.3390/cancers13236126

**Published:** 2021-12-05

**Authors:** Tomas Drizdal, Gerard C. van Rhoon, Rene F. Verhaart, Ondrej Fiser, Margarethus M. Paulides

**Affiliations:** 1 Hyperthermia Unit, Department of Radiation Oncology, Erasmus MC Cancer Institute, Dr. Molewaterplein, 3015 GD Rotterdam, The Netherlands; g.c.vanrhoon@erasmusmc.nl (G.C.v.R.); r.f.verhaart@gmail.com (R.F.V.); m.m.paulides@tue.nl (M.M.P.); 2 Department of Biomedical Technology, Faculty of Biomedical Engineering, Czech Technical University in Prague, nam. Sitna 3105, 272 01 Kladno, Czech Republic; ondrej.fiser@fbmi.cvut.cz; 3 Department of Electrical Engineering, Eindhoven University of Technology, De Rondom 70, 5612 AP Eindhoven, The Netherlands

**Keywords:** hyperthermia, head and neck, microwave applicator, specific absorption rate, water bolus, temperature prediction

## Abstract

**Simple Summary:**

Hyperthermia cancer treatment is used as an adjuvant treatment modality to standard radiotherapy and/or chemotherapy treatments. The HYPERcollar3D allows focused microwave heating to 40–44 °C in the head and neck region. A flexible bolus is placed between patient and applicator, and deionized water is circulated, to improve power transfer efficiency and allow for surface cooling. The temperature at which this water is controlled influences the temperature distribution in this target region but its influence was unknown. To understand the impact of the water bolus temperature we performed a simulation study. We experimentally established the mean heat transfer coefficient for the water bolus as 292 W m^−2^K^−1^ (range 59–520). Then, we studied the influence of the water bolus temperature on temperatures in the target region using 20 patient specific 3D models. We found that for targets located up to 20 mm from the surface (median depth), the water bolus HYPERcollar3D temperature 30 °C can be increased to 35 °C, which will increase the temperature in the target region and thus translates to overall improvements in the hyperthermia treatment quality.

**Abstract:**

During hyperthermia cancer treatments, especially in semi-deep hyperthermia in the head and neck (H&N) region, the induced temperature pattern is the result of a complex interplay between energy delivery and tissue cooling. The purpose of this study was to establish a water bolus temperature guide for the HYPERcollar3D H&N applicator. First, we measured the HYPERcollar3D water bolus heat-transfer coefficient. Then, for 20 H&N patients and phase/amplitude settings of 93 treatments we predict the T50 for nine heat-transfer coefficients and ten water bolus temperatures ranging from 20–42.5 °C. Total power was always tuned to obtain a maximum of 44 °C in healthy tissue in all simulations. As a sensitivity study we used constant and temperature-dependent tissue cooling properties. We measured a mean heat-transfer coefficient of *h* = 292 W m^−2^K^−1^ for the HYPERcollar3D water bolus. The predicted T50 shows that temperature coverage is more sensitive to the water bolus temperature than to the heat-transfer coefficient. We propose changing the water bolus temperature from 30 °C to 35 °C which leads to a predicted T50 increase of +0.17/+0.55 °C (constant/temperature-dependent) for targets with a median depth < 20 mm from the skin surface. For deeper targets, maintaining a water bolus temperature at 30 °C is proposed.

## 1. Introduction

For clinical application of deep hyperthermia (HT) in the head and neck (H&N) region, we use electromagnetic (EM) energy to heat the tumor [1,2,3]. For controlled heating of the target region to 40–44 °C we developed the HYPERcollar3D applicator consisting of 20 patch antennas operating at 434 MHz arranged in three rings [1,4]. The HYPERcollar3D exploits active phase and amplitude steering to focus the EM energy to apply effective HT to deep-seated tumors as well as those extending towards the skin surface [5]. During HT treatments, a bolus with de-mineralized water (“water bolus”) is placed between the applicator and patient surface, which is crucial to smooth irregularities of the patient’s skin surface, efficiently transfer the EM energy, and to control skin temperature, i.e., prevent hot-spots on the skin. The water bolus temperature has, however, also a great impact on the temperature of the superficial tissue layers. Since the superficial tissue can also include tumor tissue, it can be part of the target volume. A proper selection of the water bolus temperature is essential to reach the desired temperature profile in the whole target volume.

For the first generation of the HYPERcollar applicator (12 patch antennas in two rings) the water bolus temperature in clinical practice was selected and controlled at 20 °C [2,6], except when the target region extended to within 5 mm from the skin. In this case, a separate water bolus controlled at 40 °C was applied to extend the heating to the skin. However, this water bolus often was compressed partly by the large bolus and led to the undesired addition of isolating material and increased presence of EM field-distorting air inserts [7]. The temperature of 20 °C, which is also standard for deep hyperthermia treatments in the pelvic region using the Sigma-60 or Sigma-Eye applicator (Pyrexar Medical, Salt Lake City, USA), was used to ensure heat removal from the superficial layers. Moreover, as the HYPERcollar patch antennas were located within the water bolus, the allowed temperature variation was restrained, since the resonance frequency of the patch antennas shifted with the applied water bolus temperature change [8]. In contrast, the HYPERcollar3D was designed such that the patch antennas operate in an independent water compartment. This design resulted in a more stable water bolus shape, and hence a more reproducible and predictable specific absorption rate (SAR) distribution [1]. It also allows a free selection of the temperature of the water bolus connecting to the skin without compromising antenna efficiency [4]. In clinical practice it meant that the water bolus temperature for HYPERcollar3D treatments was raised to 30 °C, with the objective to increase the overall temperature within the target region and to increase patient comfort.

The purpose of this study was to assess the influence of the water bolus temperature on the resulting hyperthermia treatment quality for patients treated in the H&N region using the HYPERcollar3D applicator. As a first step, we estimated the heat-transfer coefficient of the HYPERcollar3D water bolus through the fitting of simulated with measured transient temperature profiles for a dedicated experimental setup with the water bolus in good contact with a muscle-equivalent phantom. In the second step, these estimated heat-transfer coefficients were used to calculate the full 3D temperature profiles in 20 patients using the applied clinical amplitude and phase settings from 93 treatments as well as for water bolus temperatures ranging from 20–42.5 °C. To strengthen the evidence in view of clinical implication from these 3D temperature predictions, we evaluated two different blood perfusion models. The total power was optimized in order to obtain a maximum of 44 °C in the healthy tissue for all temperature simulations. Mean temperature T50 (the temperature exceeded by 50% of the voxels of the target volume) and mean SAR in the target region (calculated using the optimized power) were evaluated as a function of water bolus heat transfer coefficient and temperature.

## 2. Methods

### 2.1. Water Bolus Experiment to Assess the Heat-Transfer Coefficient

**Figure 1:**
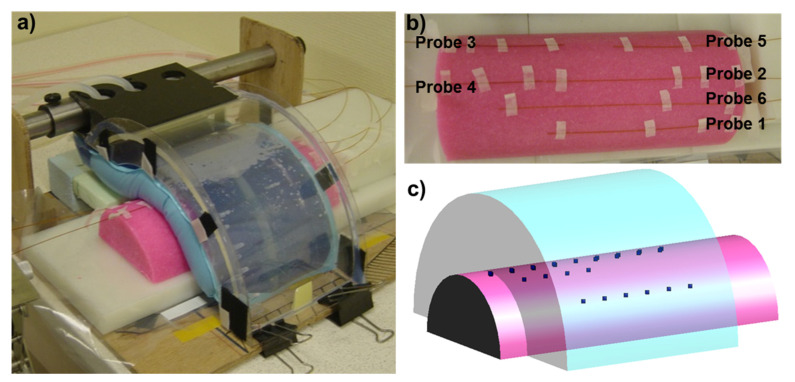
HYPERcollar3D water bolus heat transfer coefficient (**a**) measurement setup, (**b**) top view with fiber-optic probes location, (**c**) simulation SEMCAD X setup.

The HYPERcollar3D water bolus consists of left and right parts each having one inflow at the bottom and one outflow at the top, to prevent trapping of air bubbles. Both, left and right parts are each connected via plastic tubing with a diameter of 6 mm to a E4860 (Quorumtech, Laughton, East Sussex, UK) circulation unit, allowing circulation of 450 L/hour of demineralized water. The procedure described by van den Gaag et al. [9] was used to obtain the heat transfer coefficient. In this approach, the heat transfer coefficient is determined by fitting 3D temperature simulations with SEMCAD X (v. 14.8.6, Speag, Zürich, Switzerland) to the measured transient temperature profiles for a well-controlled setup, see Figure 1a. The phantom, shown in Figure 1b, was prepared following a modified recipe of Ito et al. [10] using 5.6 L of demineralized water, 54 ml of 8% formaldehyde, 31 g of salt, 401 g of poly-ethylene, 155 g of TX-151, and 168 g of agar. Figure 1b shows the location of six fiber-optic temperature measurement probes at the outer surface of the phantom with a total of 21 measurement points. The corresponding simulation setup as designed for the SEMCAD X calculations is shown in Figure 1c. For the phantom at room temperature of 19.6 °C and water bolus temperature 31.1 °C, we minimized the temperature differences by tuning the heat transfer coefficient using the f_minbnd_ function in MATLAB (v. 8.3, MathWorks, Natick, MA, USA). From this experiment, we calculated the average value of the 21 heat-transfer coefficients for each of the 21 temperature probe locations.

### 2.2. Hyperthermia Treatment Planning

**Figure 2:**
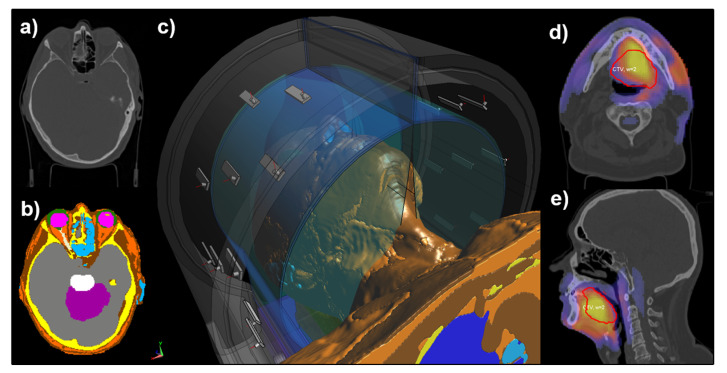
Overview of the Erasmus MC procedure of hyperthermia treatment planning for H&N hyperthermia with the HYPERcollar3D applicator. (**a**) example of axial CT slice, (**b**) corresponding segmentation in iSeg, (**c**) hyperthermia treatment planning setup in SEMCAD X, (**d**) axial and (**e**) sagittal slices of SAR with highlighted target region.

Figure 2 shows the overview of the hyperthermia treatment planning (HTP) procedure clinically applied at Erasmus MC for every H&N patient treated with the HYPERcollar3D applicator. The procedure starts with the CT scan available for radiotherapy treatment planning (Figure 2a) of which a 3D patient specific HTP model is created using automatic atlas-based segmentation [11]. This model is then imported into iSeg (v. 3.8, Zürich MedTech AG, Zürich, Switzerland) (Figure 2b), for manual verification and correction if needed. Afterwards the patient model is loaded, together with the HYPERcollar3D model, into SEMCAD X for EM field simulations (Figure 2c). The HYPERcollar3D model is positioned around the patient model in order to copy the patient position inside the device from the “test treatment” during patient HT intake. This is needed to establish specific distances between the patient surface and the applicator as well as to find the most comfortable patient position inside the HYPERcollar3D.

For the EM field simulations in this study, we used a uniform grid of 1.25 mm throughout the whole calculation domain, resulting in 70 million finite-difference time-domain cells. A harmonic 434 MHz simulation with 15 periods was calculated, typically in ten minutes per antenna, using hardware acceleration at two GTX 1080 graphical processor units. All dielectric properties were assigned following Table 1. At the end of the HTP process, all simulations were imported into VEDO, i.e., visualization tool for electromagnetic dosimetry and optimization [12]. In VEDO, we optimized the amplitude and phase antenna feeding signals in order to maximize power absorption inside the target region. Figure 2d,e show an example of axial and sagittal SAR slices from VEDO including the highlighted optimization target.

### 2.3. Temperature Modeling

The temperature distributions were calculated using standard Pennes bioheat equation [17].

$$c\rho \frac{\partial T}{\partial t}=\nabla .\left(k\nabla T\right)-SF\rho_{b}c_{b}\rho \omega \left(T-T_{b}\right)+\rho \mathrm{SAR}+\rho Q$$

where *c* (J kg^−1^K^−1^) is the specific heat capacity, *c_b_* (J kg^−1^K^−1^) the specific heat capacity of blood, *ρ* (kg m^−3^) represent the density, *k* (W m^−1^K^−1^) the thermal conductivity, *ω* (ml min^−1^kg^−1^) the blood perfusion rate, *T_b_* (K) the blood temperature, and *Q* (W kg^−1^) the metabolic heat generation. *SF* (-) is a scaling factor used for implementation of the temperature-dependent blood perfusion model of Lang [5,16,18,19]. All thermal properties were assigned using the Table 1 [13,14].

### 2.4. Thermal Tissue Property Models

To show the robustness of this study in view of clinical relevance, we applied two different temperature tissue models to see if they would lead to the same conclusions.

Constant thermal stress model: blood perfusion and thermal conductivity values for fat, muscle, and tumor were found by minimizing the difference between invasively measured and simulated temperature profiles for ten patients treated with the HYPERcollar applicator [15].

**Figure 3:**
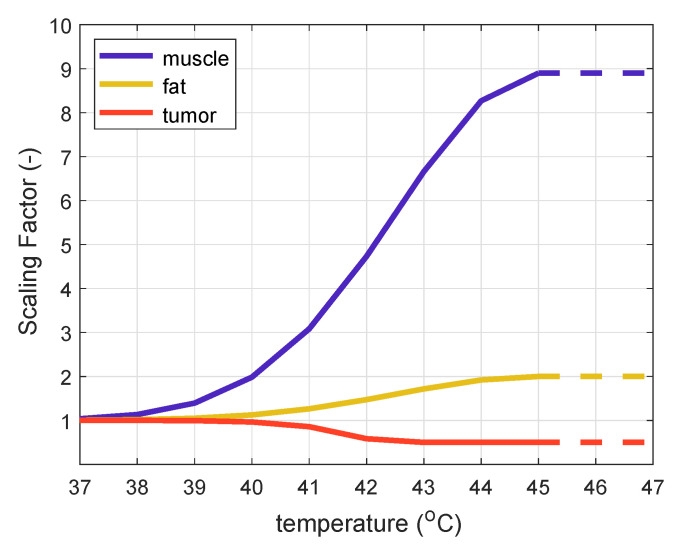
Temperature-dependent scaling factor (SF) for muscle, fat and tumor.

Temperature-dependent model: blood perfusion was piece-wise linearly increased by a factor of 2 for fat and 8.9 for muscle between 37 °C and 44 °C following the Lang model [16,18,19] (Figure 3). We followed the implementation of Lang et al. [16], and since maximum temperature in the healthy tissue did not exceed 44 °C, the perfusion values were kept constant for temperatures over 45 °C represented by dotted lines in Figure 3.

### 2.5. Impact of Water Bolus on 3D Temperature Distribution

To investigate the clinical impact of different water bolus temperature and heat transfer coefficients, we selected the first 20 patients treated using the HYPERcollar3D applicator, i.e., six females and 14 males with a mean age of 61.7 ± 12 (1 standard deviation) years. Nine patients had an oropharynx tumor, three a neck node metastasis, two a larynx tumor, two an affected parotid gland, one a sinus maxillaris tumor, one a hypopharynx tumor, one a tumor in the oral cavity, and one had a nasopharynx tumor (see Table 2). For these 20 patient models we performed full HTP modeling in which we studied the impact of 90 combinations of heat-transfer coefficient *h* (W m^−2^K^−1^) and water bolus temperature on the median target temperature T50 and mean SAR in the target region. The SAR distribution was calculated using optimized power settings from the temperature simulations, which limited the temperature in healthy tissue to maximum of 44 °C. Water bolus temperature was varied in ten steps of 2.5 °C from 20 °C to 42.5 °C and for nine different heat transfer coefficient values from minimum to maximum values of measured heat transfer coefficients.

## 3. Results

### 3.1. Water Bolus Convection Coefficient

**Figure 4:**
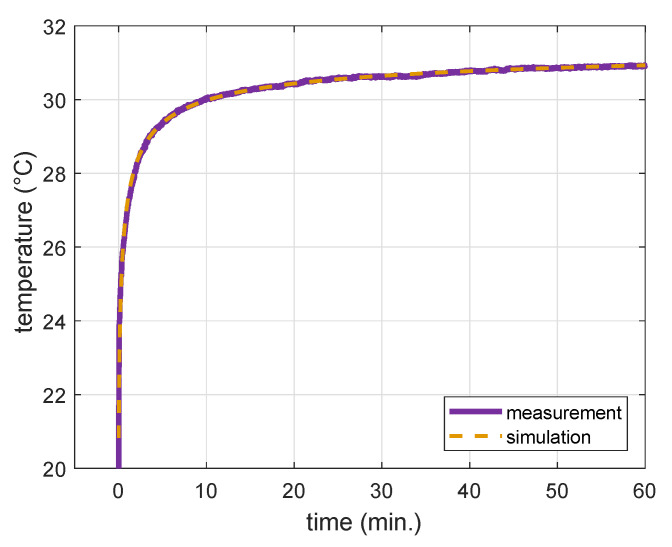
Example of fitted simulation profile using heat transfer coefficient of *h* = 308 W m^−2^K^−1^ over the measurement profile.

For the 21 measurement points the calculated mean heat transfer coefficient is *h* = 292 W m^−2^K^−1^, with a range of 59–520 W m^−2^K^−1^. Figure 4 shows an example of how closely the simulated temperature profile using *h* = 308 W m^−2^K^−1^ overlapped with the measured temperature profile. After fitting all 21 measurement points we obtained *R^2^* = 0.976 ± 0.015.

### 3.2. Impact of Water Bolus on 3D Temperature Distribution

**Figure 5:**
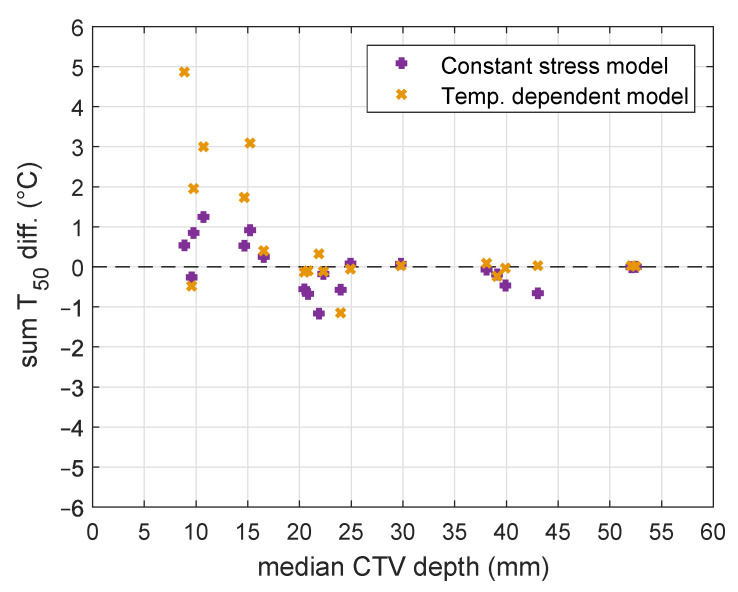
T50 mean temperature gradients for water bolus change from 20 °C to 40 °C and heat transfer coefficient of *h* = 292 W m^−2^K^−1^ as function of median CTV depths (depths of half of the CTV volumes) in all 20 patients for constant stress model and temperature-dependent model. Note that the T50 values of the two temperature models overlap for some median CTV depths.

Figure 5 shows the influence of water bolus temperature increase from 20 to 40 °C on a sum of T50 differences per patient against median CTV depths for (a) the constant thermal stress model and (b) the temperature-dependent model, using 292 W m^−2^K^−1^ for the heat transfer coefficient *h*. The sum of T50 differences were calculated for every patient as a mean value from all treatments for T50 increases/decreases when changing the water bolus temperature from 20 to 40 °C with step of 2.5 °C. We subtracted T50 for water bolus temperatures of 22.5 °C and 20 °C, which was added to the T50 difference for temperatures of 25 °C and 22.5 °C, etc. Both models predict for 6/7 patients with CTV median depth (depth of half of the CTV volume) less than 20 mm form the surface that T50 increases with water bolus temperature elevation. For deeper-seated targets (median CTV depth > 20 mm), increasing the water bolus temperature results in decreasing of T50. After this initial analysis, we added a water bolus temperature of 42.5 °C to confirm that T50 decreases for several patients at temperatures above 40 °C.

**Figure 6:**
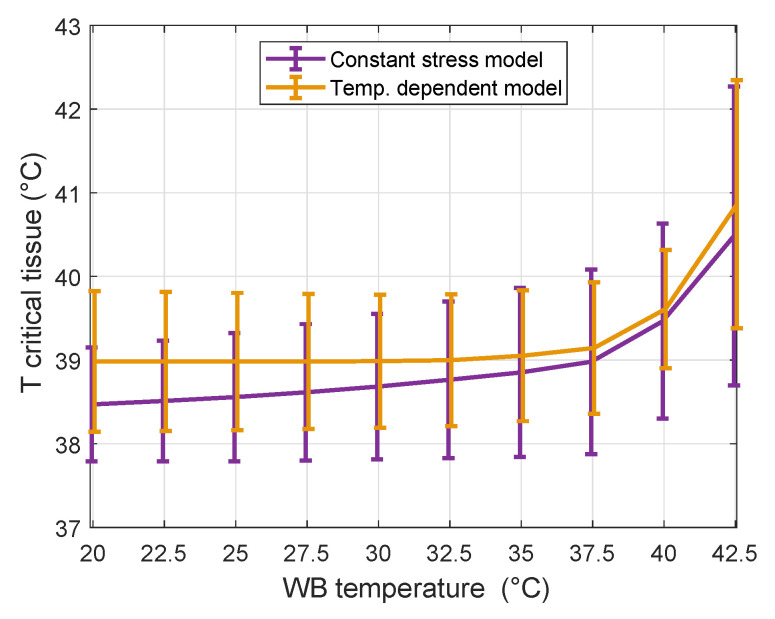
Average maximum temperature (±1 standard deviation) in critical tissues (spinal cord, brainstem and eyes) as a function of water bolus temperature and water bolus heat transfer coefficient *h* = 292 W m^−2^K^−1^.

Figure 6 shows mean of all maximum temperatures (± 1 standard deviation) in critical tissues (spinal cord, brainstem, and eyes) as a function of water bolus temperature for both studied thermal tissue models and water bolus heat transfer coefficient *h* = 292 W m^−2^K^−1^. For temperature-dependent modeling it shows that the critical tissues temperature starts to increase for water bolus temperatures above 32.5 °C. In contrast, the calculations done using the constant stress model, shows that critical tissue temperatures increase for the whole range of increasing water bolus temperatures studied. Combined results show that the water bolus temperature can safely be increased up to 35 °C without substantial temperature elevation in critical tissues.

**Figure 7:**
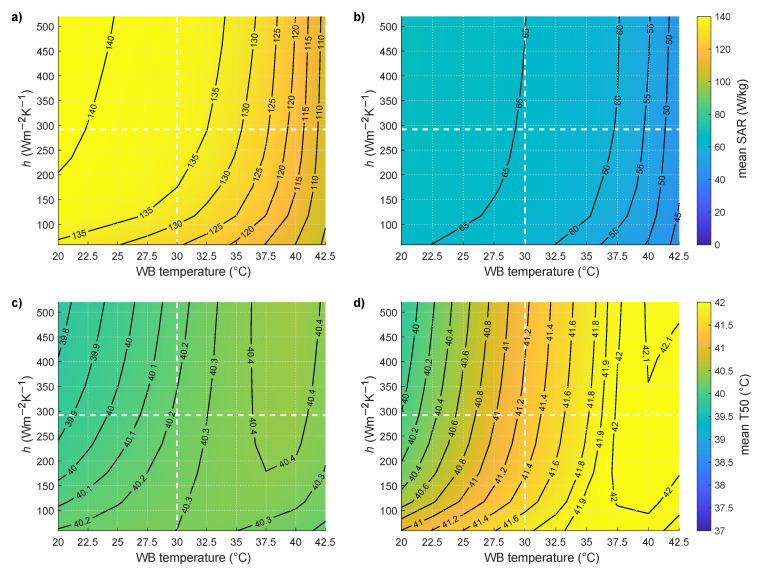
Mean SAR and mean T50 for seven patients from 34 treatments with median CTV depth less than 20 mm as a function of water bolus (WB) temperature and water bolus heat transfer coefficient (h) for (**a**,**c**) constant model and (**b**,**d**) temperature-dependent blood perfusion model.

Figure 7 shows the influence of water bolus heat transfer coefficient and temperature on mean SAR for seven patients (34 treatments) and T50 for median CTV targets less than 20 mm from the surface: Figure 7a,c for the constant stress model and Figure 7b,d for the temperature-dependent model. The mean measured heat transfer coefficient of *h* = 292 W m^−2^K^−1^ and clinical water bolus temperature of 30 °C are highlighted in Figure 7 by dotted white lines. T50 increases with higher water bolus temperature for targets less than 20 mm from the surface for both studied models. The SAR on the other hand decreases due to the lower total power required to obtain maximum 44 °C in the healthy tissue used as a threshold in all temperature simulations. Temperature-dependent modeling predicts on average an increase of ∆T50 = 1.26 °C in comparison to ∆T50 = 0.36 °C for the constant thermal stress model, when increasing the water bolus temperature from 20 to 30 °C and a mean heat transfer coefficient of *h* = 292 W m^−2^K^−1^. Additional increase of the water bolus temperature to 35 °C further increases ∆T50 = 0.52 °C for the temperature-dependent and ∆T50 = 0.15 °C for the constant thermal stress model. The mean SAR for reaching the tolerance limits decreases on average by 8% (constant stress: 140 W/kg to 131 W/kg, the temperature-dependent model: 68 W/kg to 62 W/kg) when increasing the water bolus temperature from 20 °C to 35 °C.

**Figure 8:**
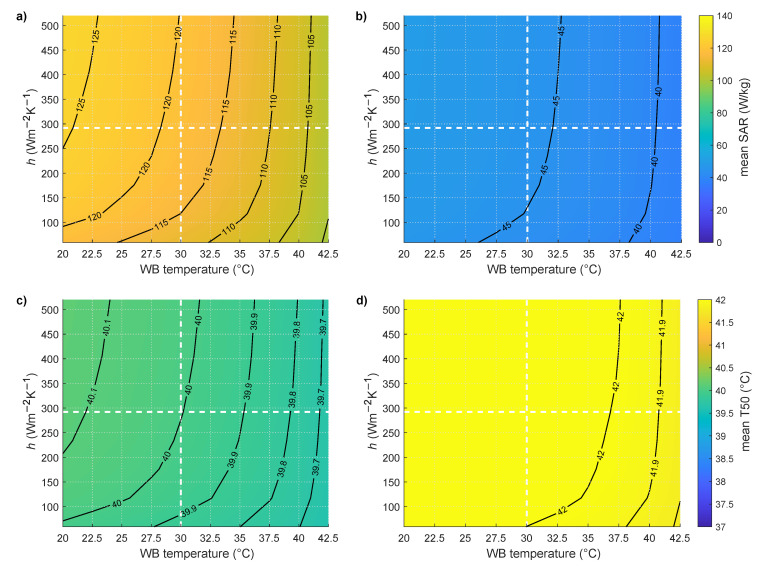
Mean SAR and mean T50 from 59 treatments with median CTV depth more than 20 mm as a function of water bolus (WB) temperature and water bolus heat transfer coefficient (h) for (**a**,**c**) constant model and (**b**,**d**) temperature-dependent blood perfusion model.

Figure 8 shows the influence of the water bolus heat transfer coefficient and temperature on the mean allowed SAR and corresponding T50 for 13 patients (59 treatments) for median CTV targets deeper than 20 mm from the surface in Figure 8a,c for the constant stress model and Figure 8b,d for the temperature-dependent model. T50 is almost constant (0.03 °C increase) for temperature-dependent and decreases by 0.11 °C for constant temperature models when increasing water bolus temperature from 20 to 30 °C. For this water bolus change, the mean SAR for reaching the tolerance limits decreases on average by 6% (constant stress: 125 W/kg to 118 W/kg, the temperature-dependent model: 49 W/kg to 46 W/kg).

## 4. Discussion

Increasing water bolus temperature improves the HT treatment quality using the HYPERcollar3D applicator for targets with median depth up to 20 mm from the surface. For these targets, increasing the HYPERcollar3D water bolus temperature from 20 °C to 30 °C increases the predicted mean T50 by 0.36 °C and 1.26 °C, calculated using the constant thermal stress and temperature-dependent models, respectively. Our analyses of the temperature in critical organs shows the possibility to increase the water bolus temperature to 35 °C, which would further increase the predicted mean T50 by 0.15 °C and 0.52 °C. For deeply situated targets (median depth >20 mm) the T50 for the constant stress model decreases with increasing water bolus temperature. For these targets, we propose to maintain a current water bolus temperature of 30 °C, which predicts to maintain the same (temperature-dependent model) or minimally decreased by 0.11 °C (constant stress model) T50 in comparison to a lower water bolus temperature of 20 °C.

For a good estimation of heat loss to the water bolus it is necessary to measure the water bolus heat transfer coefficient individually for every hyperthermia system, since it depends on various parameters such as, water bolus material, speed, and trajectory of the water flow. Our reconstructed heat transfer coefficients range from 59–520 W m^−2^K^−1^ are thus higher than the 41–320 W m^−2^K^−1^ as reported for the various water bolus sizes of Lucite cone applicator for superficial HT [9]. This is caused by a thinner water bolus material and a faster water circulation for the HYPERcollar3D water bolus. The reconstructed heat transfer coefficient might decrease further slightly when considering patient irregularities that increase the contact area of the water bolus, as indicated by the lower values found in the superficial HT for larger water boluses [9]. The reconstructed range would become smaller when a more homogenous water flow is available. A more homogenous water flow can be realized by applying multiple in- and outflow connections as for instance are used in the conformal microwave array applicator [20]. For that applicator, flow simulations were used to characterize the flow pattern inside the water bolus [21]. Despite that, we did not perform such simulations; the resemblance in temperature after adjusting heat transfer at all measurement provides good evidence that the single inflow/outflow water bolus homogenously cools across the whole water bolus area [1].

The HYPERcollar3D water bolus boundary conditions should be ideally modeled in the HTP temperature predictions with variable heat transfer coefficients changing in range from 59 to 520 W m^−2^K^−1^. In SEMCAD X it would require splitting the water bolus into several parts, each with a certain value of specific heat transfer coefficient, which is not practical in the clinical planning procedure. However, this study demonstrates that the water bolus temperature, rather than the heat transfer coefficient is the dominant factor for temperature coverage in the target region. Therefore, modeling of the water bolus as a single object with mean value of 292 W m^−2^K^−1^ is sufficient for HYPERcollar3D temperature predictions. We recommend that the water bolus heat transfer coefficient is measured specifically for each system. The current value of 292 W m^−2^K^−1^ is only valid for the water bolus used in our HYPERcollar3D system, but could be used as a first approach value for other phased array H&N systems to investigate the impact of water bolus temperature variation in in-silico studies.

Temperature T50 predictions together with the mean SAR distributions for two different temperature models reveals the necessity of further investigations into tissue cooling parameters under heat stress. We obtained a 2 °C higher T50 and a 2.6 times lower SAR when applying the temperature-dependent model in comparison to the thermal stress model, for median CTV depths above 20 mm, a water bolus heat transfer coefficient of 292 W m^−2^K^−1^ and a temperature of 30 °C. This is caused by the high perfusion values in the thermal stress model. However, to our knowledge, these data are the only tissue models extracted from clinical hyperthermia data, which we considered to be currently the best model applied for HYPERcollar3D temperature predictions. Discrete vasculature modeling has shown a potential to provide more realistic temperature predictions in the H&N region, but its application in clinical practice is still challenging due to the time required to construct even a limitedly detailed vessel model [22,23]. Hence, modeling tissue cooling is still a challenge and we certainly advise to use robust analyses, as performed in this study, to determine if findings also hold for other tissue cooling properties.

## 5. Conclusions

Based on the results of this study, we conclude that increasing the HYPERcollar3D water bolus temperature for superficially located targets (median depth < 20 mm) from 30 °C to 35 °C is safe and improve the hyperthermia treatment quality. For deeper situated targets, keeping the water bolus temperature at the currently-used 30 °C is the best option.

## Figures and Tables

**Table 1:** Dielectric properties at 434 MHz [13], thermal properties for 37 °C [14], * optimized values from ten patients treated with HYPERcollar applicator [15], the lung and internal air were in temperature simulations inactive and modeled using temperature boundary conditions. The values for tumor blood perfusion of 72.3 (mL min^−1^kg^−1^) was obtained by scaling muscle perfusion by factor of 1.85 following muscle-tumor difference at 37 °C from [16].

Tissue	*ρ*	*σ*	*ε_r_*	*k*	*c*	*ω*	*Q*
	(kg/m^3^)	(S/m)	(-)	(W/m·K)	(J/kg·K)	(ml/min·kg)	(W/kg)
Air	1.2	0	1	-	-	-	-
Blood	1050	-	-	-	3617	-	-
Bone	1908	0.09	13.1	0.32	1312	10	0.15
Brainstem	1046	1.05	55.1	0.51	3630	559	11.4
Cartilage	1099	0.6	45.1	0.49	3568	35	0.54
Cerebellum	1045	1.05	55.1	0.51	3653	763	15.5
Cerebrum	1045	0.75	56.8	0.55	3696	763	15.5
Fat	911	0.08	11.6	0.21/0.5 *	2348	32.7/255 *	0.51
Lucite	1180	0.003	2.6	-	-	-	-
Lung	394	0.38	23.6	-	-	-	-
Muscle	1090	0.8	56.7	0.49/0.4 *	3421	39.1/442.8 *	0.96
Optical nerve	1075	0.46	35	0.49	3613	160	2.5
Sclera	1032	1.01	57.4	0.58	4200	380	5.9
Spinal cord	1005	1.53	69	0.59	4047	160	2.5
Tumor/GTV	1050	0.89	59	0.51/1.5 *	3950	72.3/848 *	0
Thyroid	1050	0.89	61.3	0.52	3609	5624	87
Vitreous humor	1005	1.53	69	0.59	4047	0	0
Water	1000	0.04	78	-	-	-	-

**Table 2:** Patient details including age at the beginning of the HT treatment, gender, CTV location, median, minimum and maximum CTV depths, and amount of received HT treatments.

Patient	Age (Years)	Gender	CTV Location	CTV Median Depth (mm)	CTV Minimum Depth (mm)	CTV Maximum Depth (mm)	HT Treatments
1	23	M	nasopharynx	52.5	36.5	70.8	6
2	65	M	oropharynx	38.1	19.5	58.1	3
3	75	F	parotid gland	8.9	0.3	29.1	4
4	69	M	oropharynx	20.5	3.6	38.8	3
5	45	M	neck node metastasis	15.2	0	54.6	5
6	68	M	oropharynx	43.0	11.7	78.5	3
7	65	M	neck node metastasis	14.7	0.9	34.4	5
8	55	M	oropharynx	39.9	22.5	60.0	3
9	68	M	neck node metastasis	10.7	2.1	30.6	4
10	69	M	oropharynx	9.6	0	29.2	3
11	67	F	parotid gland	22.3	4.8	45.9	6
12	60	M	oropharynx	21.9	7.8	46.0	5
13	56	F	hypopharynx	16.5	3.1	35.9	6
14	54	F	larynx	9.8	0	28.0	7
15	75	M	oropharynx	29.8	1.9	63.2	7
16	68	M	oropharynx	39.1	26.6	57.7	3
17	72	F	oral cavity	24.9	1.2	52.6	5
18	65	M	larynx	24.0	5.7	46.6	6
19	61	M	oropharynx	20.8	2.2	60.9	5
20	53	F	sinus maxillaris	52.1	27.9	71.6	4

## Data Availability

The data presented in this study are available on request from the corresponding author.
